# Mental Health Impact and Burnout in Critical Care Staff During Coronavirus Disease 2019 Outbreak

**DOI:** 10.5152/TJAR.2022.21263

**Published:** 2022-04-01

**Authors:** Carla Gramaglia, Simona Bazzano, Eleonora Gambaro, Tiziana Cena, Danila Azzolina, Alessandro Costa, Patrizia Zeppegno, Francesco Della Corte

**Affiliations:** 1 Division of Psychiatry, Department of Translational Medicine, UPO, AOU Maggiore della Carità, Novara, Italy; 2 Department of Anaesthesia and General Intensive Care, AOU Maggiore della Carità, Novara, Italy; 3 Department of Translational Medicine, Università del Piemonte Orientale, Novara, Italy

**Keywords:** critical care, depression, ICU staff, mental health, physician wellbeing, PTSD

## Abstract

**Objective:**

The coronavirus disease 2019 outbreak exposed intensive care unit health care workers to a psychological burden. The aim of the study was to assess burnout, depression, anxiety, and post-traumatic stress symptoms in the intensive care unit staff during the pandemic period and to focus on the factors that contributed to psychological discomfort by using validated psychometric tools.

**Methods:**

This was a monocentric study developed at the end of the first emergency crisis period (May 2020). We used a custom-designed survey using SurveyMonkey. The first part of the online survey included 27 general questions (sociodemographic information, the professional role, and possible changes assigned in job tasks and duties), the second part included validated psychometric tools: Maslach Burnout Inventory, General Health Questionnaire-12 Items, Impact of Event Scale, Beck Anxiety Inventory, and Beck Depression Inventory-II. Factors independently associated with reported symptoms of mental health disorders were identified.

**Results:**

The response rate was 88%, with 95 respondents. Depressive and mild-moderate anxiety symptoms were reported in 20% and in 12% of health care workers, respectively, and half of the sample experienced moderate or severe post-traumatic stress symptoms. In total, 64% of health care workers reported high levels of burnout. General mental health problems were more frequently reported by women (*P* = .3), by those who were tested negative for the coronavirus disease 2019 buffer (*P* < .02), and by those who changed their family habits (*P* = .02) as a consequence of the pandemic. Being single or divorced (*P* = .04) was associated with the presence of depressive symptoms; vice versa, cohabiting with a partner or being married was associated with lower levels of depression. Anxious symptoms were reported in health care workers with no previous working experience in the intensive care unit.

**Conclusions:**

Health care workers experience high levels of psychological burden during the coronavirus disease 2019 pandemic. Knowing the risk factors can aid to develop strategies of observation and prevention and also strengthen the ability to be resilient to stressful situations.

## Main Points

- Depressive and mild-moderate anxiety symptoms were reported in 20% and in 12% of HCWs, respectively, and half of the sample experienced moderate or severe PTS symptoms. In total, 64% of HCWs reported high levels of burnout.
- Coronavirus disease 2019 pandemic led to an important psychological burden in HCWs in ICU with mild or moderate symptoms of anxiety, moderate or severe PTS symptoms, and knowing the risk factors can aid to develop strategies of observation and prevention.

## Introduction

On January 30, 2020, the World Health Organization declared the coronavirus disease 2019 (COVID-19) outbreak a public health emergency of international concern. From the end of December 2019 to October 2021 there have been more than 34 million confirmed cases of COVID-19 globally, including more than 1 million reported deaths.

Our hospital was one of the first in Italy to be severely and suddenly hit by the pandemic requiring a significant increase in the number of intensive care beds fully dedicated to COVID-19 patients (from 14 up to 29). Thus, initially, 5 operating rooms, with a total of 15 beds, distributed far from the usual intensive care unit (ICU), were adapted into COVID-19 ICU stations and in just 5 days, a corridor adjacent to the ICU has been adapted by modifying its structural characteristics and making it suitable for clinical assistance, homogenizing staff competencies, and patient care. To deal with the huge increase of working hours, the staff was implemented by recruiting anaesthesiologists and nurses from the ordinary surgical teams, enrolling also anaesthesia and critical care residents in their fourth and fifth postgraduate years. 

As shown in previous studies performed during severe acute respiratory syndrome, Middle East respiratory syndrome, or Ebola epidemics, health care workers (HCWs) called to action experienced an emotional burden that led to psychological consequences such as burnout, fatigue, anxiety, depression, and post-traumatic stress disorder (PTSD).^1-3^

The psychological burden on HCWs has been also highlighted in the COVID-19 outbreak. Among HCWs, intensivists were particularly exposed to stress: 10% of doctors in ICU showed depressive symptoms and approximately one-half of the intensivists presented a high level of burnout while researches suggested that PTSD in resuscitation providers had a prevalence of 10%. However, to date, the impact of the current pandemic on the psychological well-being of ICU medical and nursing staff has yet to be established.^4-6^

Based on this assumption, we investigated the psychological burden of health care workers who faced the outbreak in ICU with particular attention on burnout, depression, anxiety, and PTS symptoms and focused on the factors that contributed to psychological discomfort by using validated psychometric tools. 

## Methods

The study protocol was approved by the ethical board, and written informed consent was obtained from all subjects. We used a custom-designed survey using SurveyMonkey (San Mateo, Calif, USA), an internet program and hosting site that enabled us to develop an URL that has been copied and pasted into an email; an invitation was emailed at the end of the first emergency crisis period (end of May 2020) to the HCWs employed in the COVID ICU. The online survey was preceded by a presentation of the main objectives of the research, and HCWs were granted anonymity and required to give their informed consent to participate. Data gathering opened on May 26 and closed on June 15.

The first part of the online survey included 27 questions investigating general sociodemographic information, the professional role, and possible changes assigned in job tasks and duties during the peak of the pandemic that they normally were asked to handle during their daily work. With more detail, information was gathered about the following: sociodemographic characteristics (gender, age, and marital status), job features (role, usual place of work, length of service, increase in working hours compared to usual, mansion changed due to COVID-19, previous work experience in ICU, experience related to infection from COVID-19 (swab positivity to COVID-19 for themselves or their loved ones, physical symptoms related or not related to COVID-19 infection for themselves or their beloved), change in family habits, impact of job reorganization (perception of protection, perception of enhancement, impact on the quality of work). 

Standardized and validated self-administered measures were used for the assessment of burnout, overall health perception, distress perceived as a consequence of stressful life events, depression, and anxiety.

### Maslach Burnout Inventory-Human Services Survey for Medical Personnel

The Maslach Burnout Inventory-Human Services Survey for Medical Personnel (MBI-HSS MP) investigates the experience of occupational burnout in individuals who work with people (human services and medical professionals). Three components are identified: emotional exhaustion (EE, 9 items), which measures feelings of being emotionally overextended and exhausted at work; depersonalization (D, 5 items), which measures an unfeeling and impersonal response toward recipients of one’s service, care, treatment, or instruction; personal accomplishment (PA, 8 items) which measures feelings of competence and achievement in one’s work with people. Burnout is suggested by high EE or high D scores or by low PA ones. The Cronbach’s alpha was satisfactory for PA (alpha = 0.71) and EE (alpha = 0.85) and moderate for DP (alpha = 0.58).^7,8^

### Ge*neral Health Questionnaire-12 Items*

It is the most extensively used screening instrument for common mental disorders; higher scores on the GHQ indicate worse mental well-being.^9,10^ General Health Questionnaire-12 Items (GHQ-12) Cronbach’s alpha is 0.9.^11^

### Impact of Event Scale

The Impact of Event Scale (IES) is a 15-item self-report questionnaire designed to assess current subjective distress for any specific life event.^12,13^ It measures intrusive (intrusive thoughts, nightmares, intrusive feelings and imagery [7 items]). and avoidance symptoms (numbing of responsiveness, avoidance of feelings, situations, ideas [8 items]). The scores for the subscales range from 0 to 35 and from 0 to 40 for intrusive and avoidance symptoms, respectively. The sum of the 2 subscales is the total subjective stress score, with a cut-off point of 26 (scores > 26: moderate/severe symptoms). Both subscales have displayed acceptable reliability (alpha of 0.79 and 0.82, respectively); split-half reliability for the whole scale is 0.86.^12^

### Beck Anxiety Inventory

Each of the 21 items is rated on a 4-point scale, focusing on the experience of anxiety in the previous week. Total score ranges from 0 to 63, with the following cutoff values: 0-7 = minimal anxiety; 8-15 = mild anxiety; 16-25 = moderate anxiety; and 26-63 = severe anxiety.^14^

The Beck Anxiety Inventory (BAI) proved highly internally consistent (Cronbach’s alpha = 0.94) and acceptably reliable over an average time lapse of 11 days (*r* = 0.67).^15^

### *Beck Depression Inventory-II *(Beck and Steer, 1988)

Each of the 21 items is scored on a 4-point scale; the higher the total score, the more severe is depression; standardized cut-off values are the following: 0–13 = minimal depression; 14–19 = mild depression; 20–28 = moderate depression; 29–63 = severe depression. Beck Depression Inventory-II (BDI-II) demonstrated an overall Cronbach’s alpha of 0.90.^16,17^

### Statistical Analysis

Continuous data were synthesized as median (I and III quartiles); categorical data were summarized as percentages and absolute frequencies. The Wilcoxon-type tests were performed for continuous variables and the Pearson’s chi-square test, or Fisher’s exact test, whenever appropriate, for categorical ones.

The categorized scores were considered as endpoints. A proportional odds model was estimated for the ordinal responses with more than 2 categories. The model estimated univariable odds ratios (ORs) together with the 95% CI and the *P* values were reported. The ordinary least square estimates have been also reported for the continuous endpoints with 95% CIs. The computations were performed using the software R 4.0.2^9^ with the rms^10^ package.

### Results

The invitation was sent to 107 HCWs; 96 clicked on the link and 95 completed it, obtaining a response rate of 88% and a completion rate of 98%. Respondents’ characteristics and reports about the COVID-19 experience are presented in Table 1.

Analyzing the psychological impact of the restyling and restructuring of the workplaces, according to the opinion of 45 (n = 45, 47.9%) HCWs, the reorganization of spaces and the number of beds had been adapted taking into account the resources of available health personnel, while for 49 (n = 49, 52.1%) HCWs, it did not take in account the resources of available health personnel. A total of 36 (n = 36, 38.3%) HCWs thought that the reorganization of resuscitation in the corridor in front of the ICU had facilitated their work compared to the location in the operating room block, 41 (n = 41, 43.6%) HCWs thought the opposite, while for 17 (n = 17, 18.1%), this question was not applicable. For 75 (n = 75, 81.5%) HCWs, the reorganization of spaces and the number of beds from the operating room block to the corridor had an impact on the quality of work, while for 17 (n = 17, 18.5%), it did not. According to the opinion of 73 (n = 73, 79.3%) HCWs, the reorganization of spaces from the operating room block to the corridor had an impact on their mood about the work (vs n = 19; 20.7%): 33 (n = 33, 35.9%) HCWs felt that this reorganization made them more motivated in the work, while 59 (n = 59, 64.1%) felt that it did not. Despite the reorganization of workspaces and the number of beds, 55 (n = 55, 58.5%) HCWs perceived their work as valuable.

Table 2 shows frequencies, mean values and confidence intervals for the HCWs’ mental health outcomes assessed in the study. Table 3 includes the model estimated Univariable Odds Ratios (OR) together with the 95% confidence interval (CI), and the p-values of the self-administered questionnaires (GHQ, BDI, BAI, IES). Table 4, table 5, table 6 summarize baseline descriptive statistics of the MBI-HSS MP subscales, EE, D and PA. Table 2, 3, 4, 5, 6 report only data with p-value significative (*P* < .05).

### Discussion

Our study shows that the ICU HCWs involved in our sample experienced an important psychological burden. Most HCWs reported some (n = 35) or several (n = 48) mental health problems. Overall depressive and mild-moderate anxiety symptoms were reported in 20% and 12% of HCWs, respectively, and half of the sample (n = 45) experienced moderate or severe PTS symptoms. Regarding overall burnout, depersonalization (D) was moderate in 53 HCWs and high in 11, and the majority of HCWs reported a low level of PA at work. High scores in just 1 subscale of the MBI are enough to suggest the presence of burnout; therefore, in our sample, it can be inferred that 68 HCWs reported high levels of burnout.

Our results concerning depressive and anxiety symptoms are consistent with those by Hu et al.^18^ Available studies describe the rates of depressive symptoms ranging from 11% to 30.2% and of anxiety ranging from 14% to 46.5%.^19^^,^^20^ Nonetheless, it should be considered that the sample size is quite different in the available studies, as well as the self-administered questionnaires used.^18^,^19,21^

As far as IES scores are concerned, the study by Luceño-Moreno et al^19^ found a significantly higher rate of participants showing moderate/high scores than in our sample (83% vs 48.34%).^[Bibr b20-tjar-50-suppl1-s34]^ Overall symptoms were reported more frequently by women than by men, as in our study. More specifically, general mental health problems were more frequently reported by women, by those who were tested negative for the COVID 19 buffer, and by those who changed their family habits as a consequence of the pandemic.^19^ Furthermore, a strong association was found between being single or divorced and the presence of depressive symptoms; vice versa, cohabiting with a partner or being married was associated with lower levels of depression as also shown by Kannampallil and reported by Adams.^20,22^

Concerning anxious symptoms, higher anxiety levels (*P* = .02) are reported in HCWs with no previous working experience in ICU. Our results about the population at risk for anxious-depressive symptoms are in line with the available literature.^18-20,23^

Concerning the impact of the traumatic event (the COVID-19 pandemic), being men not having changed family habits maintaining pre-COVID-19 pandemic jobs and positivity for COVID-19 buffer emerged as protective factors, while the reorganization of spaces and the ratio among beds and manpower was a risk factor for subjective distress (OR = 2.212, CI = 1.044 to 4.6852).

Focusing on burnout, our study reports that high scores in the EE subscale of the MBI-HSS MP were more frequently associated with high levels of depersonalization (D), many psychological health problems, and low to medium levels of anxiety. Among HCWs exposed to an increase in the number of working hours during the COVID-19 pandemic, higher D levels were observed. Health care workers whose beloved had physical symptoms related to COVID-19 infection reported low or moderate D. Moderate or high D scores were observed among HCWs who changed their work habits due to the fear of falling ill with COVID-19 while the perception of being protected when working in the COVID areas for the entire period allowed on-duty staff to maintain low levels of depersonalization. 

As far as PA is concerned, it was higher in the population over 40, than in those under the age of 40. Moderate or low levels of PA were observed in HCWs who perceived that the reorganization of the spaces and the number of beds from the operating room block to the corridor had an impact on the quality of work during the COVID-19 pandemic. Unexpectedly, it turned out that the perception that one’s work was valued despite the reorganization of the spaces and the number of beds from the operating room block to the corridor during the COVID-19 pandemic was associated with moderate levels of PA.

The model-estimated univariable ORs reported in Table 4 also show that perception that one’s own work was valued despite the reorganization of the spaces and the number of beds from the operating room block to the corridor led to higher levels of D. An age of more than 40 years was associated with higher PA as the perception that one’s own work was valued despite the reorganization of the spaces and the number of beds the increase of the number of working hours and the perception of protection during the entire period of work in the COVID-19 area.

Even if we do not have a baseline measurement in our cohort, comparing data from the literature, it can be postulated that burnout levels are higher in current events than in previous periods, especially for D. A systematic review performed in 2015 reported the presence of burnout, measured with the MBI, in 30% of HCWs working in the emergency department.^24^ Also an Italian report in 2008, which analyzed the level of burnout among general practitioners with the MBI scale, highlighted the presence of medium/high EE in 32% of cases, medium/high D in 53%, and low/average PA in 32% of cases, lower than those observed in our study (moderate/high EE in n = 8, 39.36%, moderate/high D in 78.72%, low/moderate PA in 92.55%).^25^

For the analysis of burnout levels, some works use the Stanford Professional Fulfillment Index instead of the MBI scale. This Index has a single item for EE on a 7-point Likert scale (1: strongly disagree to 7: strongly agree) using the statement, “I am burned out from my work.”^20,25^ Differently, other studies used a similar scale to the current one.^18,19,21,26^

Azoulay et al^21^ analyzed burnout in a population of specialists in ICU, while Luceno-Moreno^19^ assessed different categories of HCWs; in the work by Hu,^18^ a population of nurses was studied. In these 3 studies, they observed higher percentages of HCWs experiencing lower EE (53%, 64%, and 60% respectively) but lower D values (57%, 35%, and 42%, respectively) than those observed in the current study; low PA values (67%, 91.6%, and 60.5%, respectively) were overlapping or lower than those observed in our study. The female gender and young age were factors associated with greater fragility in our study and in almost all the comparison studies analyzed.

While there are some non-modifiable risk factors (female gender and younger age), it can be argued that other factors should be addressed to reduce the risk of psychological impact in HCWs. Creating an environment that gives a sense of safety and welcome is a cornerstone of well-being, and this should particularly be considered during a pandemic when, as also shown in our study, the reorganization of spaces and the number of beds can be considered not adequate and thus create a sense of anxiety related to the event. Change in the mansion during the emergency could create a load of stress in physicians and nurses who are not normally employed in ICU. Moreover, HCWs who increase the number of working hours were more prone to develop signs of burnout. In fact, the amount of emotional workload depends, among other things, on the frequency, the intensity, and the endurance of and involvement in emotional and stressful situations, the increase in working hours, and also on the number of patients needing intensive care treatment. All of these issues represent a continuous exposure to an important emotional load, which can lead to acting against one’s own emotions with an endless struggle between true feelings and the ones suppressed, eventually causing burnout and alienation.^27^

The use of validated tools for mental health assessment, including burnout as well as anxiety, depression, PTS, and overall mental health, is a strength of this study. Moreover, the response rate we obtained is really high, even higher than in other similar studies. Furthermore, information was gathered about several sociodemographic, working habits-related, and pandemic-related variables. 

Nonetheless, some limitations should be underlined. First, the research is limited to a single center in Piedmont, a high-risk, but the restricted area, in Italy even if the one who received the highest number of patients in the region, and the sample size is small even though the response rate was excellent. 

For the cross-sectional study design of this study, it is difficult to derive causal relationships from the cross-sectional analysis. As in other similar studies, objective data about previous psychiatric disorders were not available. Moreover, in our study, we had no availability of previous measures of the psychological variables investigated.

The psychological evaluation was not conducted with a clinical interview but only through online self-report instruments, which although validated and widely used may be less precise and accurate. Finally, stress symptoms were evaluated with IES scale and not with the validated scale for the evaluation of stress symptoms (COVID-19 IES), specific to the current pandemic but not available at the time of our research. 

## Conclusions

Coronavirus disease 2019 pandemic led to an important psychological burden in HCWs in ICU with mild or moderate symptoms of anxiety, moderate or severe PTS symptoms, while depressive symptoms were rare in our sample. Regarding overall burnout, a low or moderate level was found, except the depersonalization (D) dimension, which was moderate/high. Generally, the risk factors for the development of psychological symptoms are female gender, younger age, change of work mansion, no previous experience in ICU, change in family habits, and being single or divorced. Knowing the risk factors can aid to develop strategies of observation and prevention and also strengthen the ability to be resilient to stressful situations. This concept has been extrapolated from military training but could be integrated into the training of young intensivists to give them some tools to face stressful events.^28,29^

## Figures and Tables

**Table 1.** Sociodemographic Characteristics of HCWs and Reports About COVID-19 Experience

Variable	Categories	%, n
Age (years)	>50	15 (14)
	40-50	36 (34)
	30-39	38 (36)
18-29	11 (10)
Gender	Female	67.4 (64)
Male	31.2 (31)
Marital status	Married or cohabitant	66 (62)
Single or divorced	35 (32)
Children	No	56 (52)
Yes	44 (41)
Professional title	Intensivist	15 (13)
	Anaesthetist	16 (15)
	Residents in training	18 (16)
	ICU nurse	21 (19)
	Operating room nurse	25 (25)
Social workers	4 (4)
Mansion changed due to COVID-19 emergency	No	31 (29)
Yes	69 (65)
Years of work	<5	37 (35)
	5-10	7 (7)
	11-15	17 (16)
>15	38 (36)
ICU Work experience before COVID-19 emergency	No	23 (22)
Yes	77 (72)
Change in the number of working hours during the COVID-19 emergency	Increase	69 (64)
Stable and/or decrease	31 (29)
Changes in family habits due to COVID-19	No	10 (9)
Yes	90 (84)
Positivity to COVID-19 nasal swab	No	89 (83)
Yes	11 (10)
Physical symptoms related to COVID-19	No	80.0 (76)
Yes	20.0 (19)
Family members with positivity to COVID-19 swab	No	28 (26)
Yes	72 (78)
Family members with physical symptoms related to COVID-19 infection	No	65 (61)
Yes	35 (33)
Changes in lifestyle due to fear of getting sick with COVID-19	No	9 (8)
Yes	91 (81)
Changes in lifestyle for fear of infecting a loved one	No	9 (8)
Yes	91 (82)

COVID-19, coronavirus disease 2019; HCWs, health care workers; ICU, intensive care unit.

**Table 4.** Baseline Descriptive Statistical Analysis of MBI EE

MBI EE		Low (%, n = 57)	Moderate (%, n = 29)	High (%, n = 8)	Combined (%, n = 94)	*P*
D categorical (n = 94)	Low	30 (17)	10 (3)	0 (0)	21 (20)	<.001
	Moderate	63 (36)	52 (15)	25 (2)	56 (53)	
High	7 (4)	38 (11)	75 (6)	22 (21)
GHQ categorical (n = 93)	No problem	18 (10)	0 (0)	0 (0)	11 (10)	.035
	Some problems	41 (23)	34 (10)	25 (2)	38 (35)	
Many problems	41 (23)	66 (19)	75 (6)	52 (48)
BAI categorical (n = 92)	Low	93 (52)	83 (24)	71 (5)	88 (81)	.006
	Moderate	7 (4)	17 (5)	14 (1)	11 (10)	
High	0 (0)	0 (0)	14 (1)	1 (1)

MBI, Maslach Burnout Inventory; EE, emotional exhaustion; D, depersonalization; GHQ, General Health Questionnaire; BAI, Beck Anxiety Inventory.

**Table 2.** Frequencies, Mean Values, and CI for GHQ, BDI, BAI, IES, and MBI-HSS MP

Variable (n)		n	%	Mean Value	CI (Min-Max Value)
GHQ	No problem	10	10.75	12	10-14
	Some problems	35	37.63	17	17-18
	Several problems	48	51.61	23	21-27
Total	93	100	19	17-23
BDI	Minimal	71	78.89	6	2-8.5
	Mild	11	12.22	16	15.5-18.5
	Moderate	8	8.89	23	20.8-24.8
Total	90	100	7	3-13
BAI	Low	81	88.04	6	2-9
	Moderate	10	10.87	26.5	24.2-31.2
	High	1	1.09	39	39-39
Total	92	100	7	2.8-13
IES	Subclinical	19	20.65	5	3-6
	Mild	28	51.09	16.5	11.8-20
	Moderate	25	27.17	33	30-40
	Severe	20	21.17	54.5	49-62.2
Total	92	100	25	9.8-41
MBI EE	Low	57	60.64	12	9-15
	Moderate	29	30.85	21	19-23
	High	8	8.51	36	32-41
Total	94	100	16	11-21
MBI D	Low	20	21.28	3	1.8-4.0
	Moderate	53	56.38	8	7-10
	High	21	22.34	16	14.18
Total	94	100	8	6-12
MBI PA	High	7	7.45	43	42-45
	Moderate	27	28.72	36	35-37
	Low	60	63.83	28	26-31
Total	100	100	32	27-32

MBI, Maslach Burnout Inventory; EE, emotional exhaustion; D, depersonalization; PA, personal accomplishment; BAI, Beck Anxiety Inventory; BDI, Beck Depression Inventory; IES, Impact of Event Scale; GHQ, General Health Questionnaire.

MBI EE: Low ≤17, Medium 18-29, High ≥30.

MBI D: Low ≤05, Medium 6-11, High ≥12.

MBI PA (reverse score): High ≥40 (low burnout), Medium 35-39 (medium burnout), High ≤34 (low burnout).

Beck Anxiety Inventory: minimum 0-21, medium 22-35, high ≥ 36.

**Table 3.** The Model Estimated Univariable OR, 95% CI, and the *P* Values

Higher Scores GHQ, BDI, BAI, IES		OR	95% CI	*P*
**GHQ**	Gender, male	0.389	0.168-0.899	.03
	Positivity to COVID-19 buffer	0.209	0.054-0.811	.02
Family habits did not change due to the COVID-19 emergency	0.208	0.058-0.747	.02
**BDI**	Civil Status: single or divorced versus cohabitant or married	29.341	10.474-82.191	.04
**BAI**	Absence of work experience before the COVID-19 pandemic in intensive care	46.563	12.666-17.117	.02
**IES** **-**	Gender, male	0.22881	0.09852-0-.53138	<.001
	Family habits did not change due to the COVID-19 emergency	0.093969	0.021812-0.40483	<.001
	Mansion changed due to COVID-19 emergency	0.454	0.202-10.164	.05
	Positivity to COVID-19 buffer	0.275	0.076-0.995	.05
Reorganization of the spaces and the number of beds adequate taking into account the resources of health personnel available	2.2121	1.044-4.6852	.04

GHQ, General Health Questionnaire; BDI, Beck Depression Inventory; BAI, Beck Anxiety Inventory; IES, Impact of Event Scale; OR, odds ratio.

**Table 5.** Baseline Descriptive Statistical Analysis of MBI D

MBI D		Low (%, n = 20)	Moderate (%, n = 53)	High (%, n = 21)	Combined (%, n = 94)	*P*
Change in the number of working hours during the COVID-19 emergency (n = 94)	Increase	40 (8)	72 (38)	90 (18)	69 (64)	.002
Stable and/or decrease	60 (12)	28 (15)	10 (2)	31 (29)
Loved ones with physical symptoms related to COVID-19 infection (n = 94)	No	90 (18)	55 (29)	65 (13)	65 (60)	.019
Yes	10 (2)	45 (24)	35 (7)	35 (33)
Changes in lifestyle due to the fear of getting sick with COVID-19 (n = 90)	No	0 (0)	17 (8)	0 (0)	9 (8)	.023
Yes	100 (20)	83 (40)	100 (21)	91 (81)
Perception of protection while working in the COVID-19 area for the entire period (n = 94)	No	20 (4)	55 (29)	67 (14)	50 (47)	.007
Yes	80 (16)	45 (24)	33 (7)	50 (47)
EE categorical (n = 94)	Low	85 (17)	68 (36)	19 (4)	61 (57)	<.001
	Moderate	15 (3)	28 (15)	52 (11)	31 (29)	
High	0 (0)	4 (2)	29 (6)	9 (8)

COVID-19, coronavirus disease 2019; MBI, Maslach Burnout Inventory; EE, emotional exhaustion; D, depersonalization,

**Table 6.** Baseline Descriptive Statistics of MBI PA

MBI PA		High (%, n = 7)	Moderate (%, n = 27)	Low (%, n = 60)	Combined (%, n = 94)	*P*
Age (n = 95)	40+	86 (6)	63 (17)	42 (25)	51 (48)	.03
18-39	14 (1)	37 (10)	58 (35)	49 (46)
Impact of the reorganization of spaces and the number of beds, blocking of operating rooms on the quality of work during the COVID-19 pandemic (n = 92)	No	57 (4)	8 (2)	19 (11)	18 (17)	.011
Yes	43 (3)	92 (24)	81 (48)	82 (75)
Perception of the enhancement of one’s work despite the reorganization of the beds in the operating rooms during the COVID-19 pandemic (n = 94)	No	43 (3)	22 (6)	50 (30)	41 (39)	.052
Yes	57 (4)	78 (21)	50 (30)	59 (55)

COVID-19, coronavirus disease 2019; MBI, Maslach Burnout Inventory; PA, personal accomplishment.
